# Beyond Wilson–Cowan dynamics: oscillations and chaos without inhibition

**DOI:** 10.1007/s00422-022-00941-w

**Published:** 2022-09-05

**Authors:** Vincent Painchaud, Nicolas Doyon, Patrick Desrosiers

**Affiliations:** 1 Department of Mathematics and Statistics, McGill University, Sherbrooke Street West, Montreal, QC H3A 0B6 Canada; 2 Départment de Mathématiques et de Statistique, Université Laval, Avenue de la Médecine, Quebec City, QC G1V 0A6 Canada; 3 Départment de Physique, de Génie Physique et d’Optique, Université Laval, Avenue de la Médecine, Quebec City, QC G1V 0A6 Canada; 4 CERVO Brain Research Center, Avenue d’Estimauville, Quebec City, QC G1E 1T2 Canada; 5 Centre Interdisciplinaire en Modélisation Mathématique de l’Université Laval, Avenue de la Médecine, Quebec City, QC G1V 0A6 Canada

**Keywords:** Biological neural networks, Wilson–Cowan model, Dynamical systems, Markov chains, Chaos

## Abstract

Fifty years ago, Wilson and Cowan developed a mathematical model to describe the activity of neural populations. In this seminal work, they divided the cells in three groups: active, sensitive and refractory, and obtained a dynamical system to describe the evolution of the average firing rates of the populations. In the present work, we investigate the impact of the often neglected refractory state and show that taking it into account can introduce new dynamics. Starting from a continuous-time Markov chain, we perform a rigorous derivation of a mean-field model that includes the refractory fractions of populations as dynamical variables. Then, we perform bifurcation analysis to explain the occurrence of periodic solutions in cases where the classical Wilson–Cowan does not predict oscillations. We also show that our mean-field model is able to predict chaotic behavior in the dynamics of networks with as little as two populations.

## Introduction

Differential equations have been successfully used to model the activity of neurons for more than a century now, ever since the works of Lapicque (1907). One of the most important examples of such a model, published fifty years ago by Wilson and Cowan (1972), describes the average firing rates of coupled neural populations using a set of ordinary differential equations. This model, of significant historical importance, has been the starting point for many extensions and is still highly relevant today (Bressloff et al. 2016; Chow and Karimipanah 2020; Cowan et al. 2016; Destexhe and Sejnowski 2009; Wilson and Cowan 2021). An important achievement of this model was to predict oscillations and bistability in the neural activity of a network made of an excitatory and an inhibitory population.

Inhibition is a key ingredient in the dynamics of the Wilson–Cowan model. Indeed, it is well-known that their equations for a pair of populations can only predict oscillatory solutions if one of them is excitatory, while the other is inhibitory (Ermentrout and Terman 2010, Sect. 11.3.2), forming a so-called Wilson–Cowan oscillator. The model may also lead to more complicated dynamical behavior in the presence of inhibition. For instance, chaotic behaviors have been shown to arise from their equations, for example by Borisyuk et al. (1995) and by Maruyama et al. (2014), but only—at least to our knowledge—in systems of at least two coupled Wilson–Cowan oscillators. Thus, inhibition plays a crucial role in Wilson–Cowan’s model in the formation of oscillatory and chaotic behaviors, which have both been observed experimentally for a long time in the activity of neural networks and are thought to play important biological roles (Buzsáki 2006; Breakspear 2017; Rabinovich and Abarbanel 1998). In similar models as well, the role of inhibition is crucial for chaotic behavior to arise (Fukai and Shiino 1990; Sompolinsky et al. 1988). While it has been demonstrated experimentally that inhibition is important to several types of oscillations in neural networks (Bartos et al. 2007; Whittington et al. 2000), it does not explain all oscillatory behavior. For example, it has been shown in experiments that excitatory neurons in the pre-Bötzinger complex can exhibit oscillatory behavior (Butera et al. 1999a, b; Duan et al. 2017). Another example of this are theta oscillations (Buzsáki 2002; Buzsáki and Draguhn 2004), which are thought to arise as a result of the activity of excitatory neurons only (Budd 2005; Chagnac-Amitai and Connors 1989). Thus, there are still oscillatory behaviors that the classical model cannot explain.

One of the key steps in the construction of Wilson–Cowan’s model is a time coarse graining, which has the effect of setting the refractory fraction of a neural population as always proportional to its active fraction. Wilson and Cowan originally argued that at least when the parameters of the model have physiologically reasonable values, this should not have any important impact on the model. Later on, others even argued that the refractory period of neurons should not have an impact on the dynamics, and chose to neglect it completely (Curtu and Ermentrout 2001; Ermentrout and Terman 2010, Sect. 11.3). However, it has been noticed experimentally that refractoriness can have an impact on neuronal activity. For instance, Berry and Meister (1998) have shown that a longer refractory period in neurons improves the precision in the response of ganglion cells, suggesting that refractoriness improves neural signaling. Similarly, Avissar et al. (2013) have shown that refractoriness enhances precision in the timing and synchronization of neurons’ spikes, which once again suggests that it makes neurons more precise. Refractoriness is also known to be related to oscillations in neural networks, since it can help neurons to synchronize their spikes (Sanchez-Vives and McCormick 2000; Wiedemann and Lüthi 2003). In theoretical works as well, it has recently been suggested that the refractory state of neurons could be essential in some cases to provide a complete description of the dynamics of a biological neural network (Rule et al. 2019; Weistuch et al. 2021).

In this paper, we propose a simple extension of Wilson–Cowan’s classical model where the refractory state is explicitly considered with the same importance as the active and sensitive states. Indeed, using a different approach than Wilson–Cowan, we derive a dynamical system closely related to their original model, in the sense that it includes it as a subsystem. Then, we show that our dynamical system can predict oscillations and chaotic behaviors in the activity of neural networks of excitatory populations. This contrasts with the original Wilson–Cowan model, in which inhibition plays a crucial role for such phenomena to arise.

First, in Sect. 2, we present an explicit construction of the model. We start by defining a continuous-time Markov chain to describe the evolution of a large network’s state in a way that mimics the behavior of biological neurons. The resulting stochastic process is similar to one already proposed—but not extensively studied—by Cowan (1990), and is also reminiscent of a similar process proposed by Zarepour et al. (2019). Then, we reduce this Markov chain, which describes the evolution of the network’s state from a microscopic point of view, to a dynamical system of small dimension that describes the dynamics from a macroscopic point of view. To do so, we split the network into a small number of neural populations, and we obtain a dynamical system that describes the evolution of the average active and refractory fractions of each population.

Then, in Sect. 3, we study the relationship between our model and Wilson–Cowan’s classical model. In fact, we show that Wilson–Cowan’s dynamical system can be seen as a subsystem of ours. We then argue that the simplification of our model to Wilson–Cowan’s is not trivial. Indeed, the domain to which corresponds the subsystem is not invariant by the flow of the full dynamical system, so that in particular, it cannot be an attracting set.

Finally, in Sect. 4, we present a detailed study of three examples where our model succeeds in predicting the qualitative dynamical behavior of the underlying Markov chain, while the classical Wilson–Cowan model fails to do so. In particular, the first example shows that our model allows oscillations in the activity of a single excitatory population, and the second shows that it allows chaotic behavior in the activity of a pair of excitatory populations.

## The model

We consider a network of $N$ neurons labeled with integers from $1$ to $N$. Links between neurons are described by a weight matrix $W \in {\mathbb {R}}^{N\times N}$ whose element $W_{jk}$ represents the weight of the connection from neuron $k$ to neuron $j$. If neuron $k$ is excitatory, then $W_{jk} > 0$, and if it is inhibitory, then $W_{jk} < 0$.

Our goal is to build an approximate macroscopic description of the network’s dynamics starting from a precise microscopic description. To do so, we consider a partition ${\mathscr {P}}$ of the set of neurons $\{1, \ldots , N\}$. Each element $J \in {\mathscr {P}}$ then represents a population of the network, that is, a set of neurons that share similar properties in a sense that will be made precise later. We will start by defining a continuous-time Markov process to provide a precise description of the evolution of the network’s state, and then we will construct a mean-field model to describe the evolution of the population’s macroscopic states.

### The microscopic model

#### Stochastic process

In order to model biological neurons, we assume that neurons can take three states:

- 0 : the *sensitive* state,
- 1 : the *active* state,
- *i* : the *refractory* state,

where $i$ denotes the imaginary unit. The active state is that of a neuron undergoing an action potential. Following an action potential, neurons typically enter a hyperpolarized state during which another action potential cannot occur even if the neuron receives a stimulus that would be otherwise sufficient to trigger spiking (Purves et al. 2018). This state is what we call the refractory state. When a neuron is neither active nor refractory, we say that it is sensitive, as if it receives a high enough input it can spike in response.

We see the transitions between these states as random: a sensitive neuron activates at a rate that increases nonlinearly with its input, and then gets to the refractory state and then back to the sensitive state at constant rates. This intuitive process describes the evolution of the whole network’s state, and is defined rigorously as a continuous-time Markov chain $\{X_t\}_{t\ge 0}$ taking values in the state space $E \,{:}{=}\,\{0,1,i\}^N$.

**Fig. 1:**
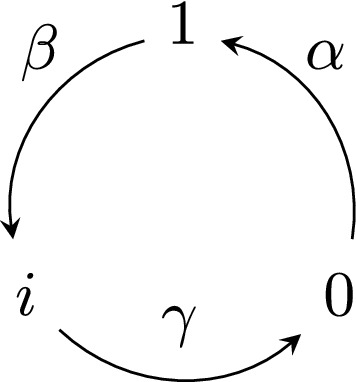
Allowed transitions between states of neurons and corresponding characteristic rates

For each neuron $j$, let $\alpha _j, \beta _j, \gamma _j > 0$. These parameters characterize the transition rates from a state to another as shown in Fig. 1. While $\beta _j$ and $\gamma _j$ both describe the actual transition rates, $\alpha _j$ rather represents the activation rate of $j$ if it is given an infinite excitation. Indeed, we assume a soft threshold dynamics: the activation rate is given by the function

$$\begin{aligned} a_j(x) \,{:}{=}\,\alpha _j F_J\left( \sum _{k=1}^N W_{jk} \mathrm{Re}x_k + Q_J \right) , \end{aligned}$$

where $F_J:{\mathbb {R}} \rightarrow [0,1]$ is a function of the neuron’s input, $Q_J \in {\mathbb {R}}$ is an input in $J$ that is external to the network, and $J$ is the population to which belongs $j$. Here, we assume that $F_J$ is a continuous and increasing function that tends to $1$ at infinity. Biologically speaking, $\beta _j$ and $\gamma _j$ can be interpreted as the inverse of the average times neuron $j$ spends in the active and refractory states, respectively. Thus, for example, a higher value for $\beta _j$ than for $\gamma _j$ translates the idea that the duration of an action potential is less than the refractory period. However, the interpretation of $\alpha _j$ is less direct. Indeed, $\alpha _j$ is the activation rate of neuron $j$ only when it is given an infinite input. Hence, the inverse of $\alpha _j$ cannot be seen directly as the average time neuron $j$ spends in the sensitive state; this time depends on the activity of the whole network. Moreover, the average and maximal firing rates vary greatly from situation to situation or according to the neural type (Roxin et al. 2011; Wang et al. 2016). This implies that the model can be relevant for a wide range of values of $\alpha _j$.

From the rates associated with the state transitions of single neurons, we can now define a generator for the Markov chain $\{X_t\}_{t\ge 0}$, which will allow to define the process correctly. This generator is a matrix $M$ indexed over the state space $E$ whose entry $m(x,y)$ gives the transition rate from a state $x$ to another state $y$. We define this rate as

$$\begin{aligned} m(x,y) \,{:}{=}\,\sum _{j=1}^N m_j(x,y) \smash {\prod _{\begin{array}{c} k=1 \\ k \ne j \end{array}}^N} \delta _{x_ky_k}, \end{aligned}$$

where $\delta _{x_ky_k}$ is a Kronecker delta and where

$$\begin{aligned} \begin{aligned} m_j(x,y) \,{:}{=}\,a_j(x)&(1 - |x_j|) \bigl (\mathrm{Re}y_j - (1 - |y_j|) \bigr ) \\ {} + \beta _j&\mathrm{Re}(x_j) (\mathrm{Im}y_j - \mathrm{Re}y_j) \\ {} + \gamma _j&\mathrm{Im}(x_j) \bigl ( (1 - |y_j|) - \mathrm{Im}y_j \bigr ). \end{aligned} \end{aligned}$$

To make sense of $m_j(x,y)$, recall that a component $x_j$ of a state vector $x \in E$ is either $0$, $1$ or $i$, so that exactly one of $\mathrm{Re}x_j$, $\mathrm{Im}x_j$ and $1 - |x_j|$ is $1$ while the others are $0$.

Now, it is a simple calculation to see that the matrix $M \,{:}{=}\,\{m(x,y) : x,y \in E\}$ is the generator of a continuous-time Markov chain. Thus, it follows from the Kolmogorov extension theorem (for details, see e.g., the books by Doob 1990 or Norris 1997) that a probability measure ${\mathbb {P}}$ exists on the space $(E,2^E)^{[0,\infty )}$ such that for any $x,y \in E$, as $\Delta t \downarrow 0$,

$$\begin{aligned} {{\mathbb {P}}}\big [{X_{t+\Delta t} = y \vert X_t = x}\big ] = \delta _{xy} + m(x,y) \Delta t + o(\Delta t). \end{aligned}$$

In particular, if $X_t^j$ denotes the $j$th component of $X_t$ and if $x$ denotes a state with $x_j = 0$, then

1a $$\begin{aligned} {{\mathbb {P}}}\big [{X_{t+\Delta t}^j = 1 \vert X_t = x}\big ]&= a_j(x)\Delta t + o(\Delta t), \end{aligned}$$

while in general

1b $$\begin{aligned} {{\mathbb {P}}}\big [{X_{t+\Delta t}^j = i \vert X_t^j = 1}\big ]&= \beta _j\Delta t + o(\Delta t), \end{aligned}$$

1c $$\begin{aligned} {{\mathbb {P}}}\big [{X_{t+\Delta t}^j = 0 \vert X_t^j = i}\big ]&= \gamma _j\Delta t + o(\Delta t), \end{aligned}$$

and the transitions $0 \mapsto i$, $i \mapsto 1$ and $1 \mapsto 0$ all have $o(\Delta t)$ rates.

In principle, the system is then completely described, since the transition probabilities ${{\mathbb {P}}}\big [{X_{t+\Delta t} = y \vert X_t = x}\big ]$ for $x,y \in E$ can be obtained from the solution of the Kolmogorov forward equation

2 $$\begin{aligned} {\dot{P}}(t) = P(t)M, \end{aligned}$$

where the dot denotes a derivative. Indeed, the solution $P(t)$ of (2) is the matrix whose entries are the probabilities that the system makes a transition from a state to another during an interval of time $t$. More explicitly, the transition probability ${{\mathbb {P}}}\big [{X_{t+\Delta t} = y \vert X_t = x}\big ]$ is equal to the element $x,y$ of $P(\Delta t)$. However, since there are $3^N$ possible states in $E$, this differential equation is enormous when the network has a large number of neurons, so that it cannot be studied directly in practice.

#### Dynamical system

Now, we want to use the stochastic process constructed above to obtain a macroscopic description of the evolution of the network’s state, in the form of a dynamical system. To do this, we first introduce functions $p_j, q_j, r_j :[0,\infty ) \rightarrow [0,1]$ given by

3a $$\begin{aligned} p_j(t)&\,{:}{=}\,{{\mathbb {P}}}\big [{X_t^j = 1}\big ], \end{aligned}$$

3b $$\begin{aligned} q_j(t)&\,{:}{=}\,{{\mathbb {P}}}\big [{X_t^j = 0}\big ], \end{aligned}$$

3c $$\begin{aligned} r_j(t)&\,{:}{=}\,{{\mathbb {P}}}\big [{X_t^j = i}\big ]. \end{aligned}$$

Since $X_t^j$ takes values in $\{0,1,i\}$, it is easy to see that $p_j + q_j + r_j \equiv 1$ and that

$$\begin{aligned} p_j(t)&= {{\mathbb {E}}}\big [{\mathrm{Re}X_t^j}\big ], \\ q_j(t)&= {{\mathbb {E}}}\big [{1 - |X_t^j|}\big ], \\ r_j(t)&= {{\mathbb {E}}}\big [{\mathrm{Im}X_t^j}\big ], \end{aligned}$$

where ${\mathbb {E}}$ denotes the expectation with respect to ${\mathbb {P}}$. Using these relations, it is possible to find expressions for the derivatives of these variables. Indeed, with $\Delta t > 0$,

$$\begin{aligned} p_j(t + \Delta t)&= {{\mathbb {P}}}\big [{X_{t+\Delta t}^j = 1}\big ] \\&= \sum _{x\in E} {{\mathbb {P}}}\big [{X_{t+\Delta t}^j = 1 \vert X_t = x}\big ] {{\mathbb {P}}}\big [{X_t = x}\big ]. \end{aligned}$$

Using the transition rates introduced earlier, we get from (1) that

$$\begin{aligned} \begin{aligned} p_j(t + \Delta t)&= \sum _{x\in E} \Bigl (\mathrm{Re}(x_j)(1 - \beta _j \Delta t) \\&\quad + \bigl ( 1 - |x_j| \bigr ) a_j(x) \Delta t + o(\Delta t) \Bigr ) {{\mathbb {P}}}\big [{X_t = x}\big ]\\&= (1 - \beta _j \Delta t) p_j(t) \\&\quad + \Delta t {\mathbb {E}}\big [\bigl ( 1 - |X_t^j| \bigr ) a_j(X_t)\big ] + o(\Delta t). \end{aligned} \end{aligned}$$

Taking $\Delta t \rightarrow 0$, it follows that

4a $$\begin{aligned} {\dot{p}}_j(t)&= - \beta _j p_j(t) + {\mathbb {E}}\big [\bigl ( 1 - |X_t^j| \bigr ) a_j(X_t)\big ]. \end{aligned}$$

Using the same method, we also find that

4b $$\begin{aligned} {\dot{q}}_j(t)&= - {\mathbb {E}}\big [\bigl ( 1 - |X_t^j| \bigr ) a_j(X_t)\big ] + \gamma _j r_j(t), \end{aligned}$$

4c $$\begin{aligned} {\dot{r}}_j(t)&= - \gamma _j r_j(t) + \beta _j p_j(t). \end{aligned}$$

At first glance, one might think that at this point, the dimension of the system has been reduced from $3^N$ to $2N$. However, the system (4) is not closed, in the sense that the derivatives of $p_j$, $q_j$ and $r_j$ are not given as functions of the same variables, but rather involve other expectations. Hence, the Kolmogorov forward equation (2) is still needed to solve (4).

### The macroscopic model

To study the network’s dynamics from a macroscopic point of view, we introduce for each population $J \in {\mathscr {P}}$ and each $t \ge 0$ the random variables

5a $$\begin{aligned} A_t^J&\,{:}{=}\,\frac{1}{|J|} \sum _{j\in J} \mathrm{Re}X_t^j, \end{aligned}$$

5b $$\begin{aligned} R_t^J&\,{:}{=}\,\frac{1}{|J|} \sum _{j\in J} \mathrm{Im}X_t^j, \end{aligned}$$

5c $$\begin{aligned} S_t^J&\,{:}{=}\,\frac{1}{|J|} \sum _{j\in J} \bigl ( 1 - |X_t^j| \bigr ), \end{aligned}$$

which can be understood as state variables for populations. Thus, the expected values of these variables describe the expected behavior of the network from a macroscopic point of view. We will use the system (4) to find a dynamical system that describes the expected behavior of the network in that sense.

Since it drastically simplifies the reduction of (4) to the macroscopic point of view, we assume that all parameters (the weights and transition rates) are constant over populations. However, we stress that this is not necessary if populations are large: we could instead assume that parameters are independent random variables, identically distributed over populations, and the resulting macroscopic model would be the same. This more general approach, which is also much more technical, is discussed in “Appendix A.”

Using the assumption described above, we see that the input in a neuron $j$ of a population $J$ becomes

$$\begin{aligned} \begin{aligned} \sum _{k=1}^N W_{jk}&\mathrm{Re}X_t^k + Q_J \\&= \sum _{K\in {\mathscr {P}}} \sum _{k\in K} W_{JK} \mathrm{Re}X_t^k + Q_J \\&= \sum _{K\in {\mathscr {P}}} |K| W_{JK} A_t^K + Q_J, \end{aligned} \end{aligned}$$

where we replaced $W_{jk}$ with $W_{JK}$ for $k \in K$, as we assume that weights are constant over populations. To simplify notation, we will write such an input as

$$\begin{aligned} B_t^J \,{:}{=}\,\sum _{K\in {\mathscr {P}}} c_{JK} A_t^K + Q_J \quad \text {with}\quad c_{JK} \,{:}{=}\,|K| W_{JK}. \end{aligned}$$

It is then easy to obtain expressions for the derivatives of the average macroscopic state variables 6a6b6c Indeed, if $j \in J$, then $a_j(X_t) = \alpha _J F_J(B_t^J)$, so it directly follows from (4) that 7a7b7c where we replaced $\alpha _j$ with $\alpha _J$ and followed the same pattern for other transition rates.

Finally, to close the above dynamical system, we add the mean-field assumption and we neglect covariances between state variables. Thus, we obtain the mean-field dynamical system 8a8b8c where .

Remark that for each $J$, one of the equations (8) is redundant since . Hence, the above dynamical system has dimension $2n$, where $n$ is the number of populations of the network. Moreover, the variables ,  and  must be contained in $[0,1]$ to make sense. Therefore, the system (8) can be studied using only the active and refractory fractions of populations, on the domain9This domain enjoys a simple invariance property.

#### Proposition 1

The domain ${\mathscr {D}}_{n}$ is invariant by the flow of the dynamical system (8).

#### Proof

Recall that all transition rates $\alpha _J, \beta _J, \gamma _J$ are poitive and that all functions $F_J$ are nonnegative. If  is a point at the boundary of ${\mathscr {D}}_{n}$, then one of the fractions ,  or  is zero at $Y$ for some population $J$; call this fraction . Then, it is clear from the equations (8) that the derivative of  must be nonnegative at $Y$, so that the vector field corresponding to the dynamical system is directed inward ${\mathscr {D}}_{n}$ at $Y$. $\square$

This invariance property is crucial to the meaning of solutions of the mean-field dynamical system (8). Indeed, for the variables ,  and  to represent proportions of neurons, all of them must remain in the interval $[0,1]$ at all times. Thus, Proposition 1 confirms that it is always possible to interpret the components of a solution of (8) as proportions of neurons in each of the three states, as long as the initial state can also be interpreted in this way. In other words, any solution of (8) that starts from a physiologically meaningful initial state continues to carry a physiological interpretation at all times.

## Relationship with the Wilson–Cowan model

The dynamical system (8) can be seen as a generalization of the classical system introduced by Wilson and Cowan (1972). Indeed, both our model and Wilson–Cowan’s describe the activities of populations of a biological neural network. Wilson–Cowan’s classical equations, which are formulated for a network split into an excitatory and an inhibitory population, are given by

10a $$\begin{aligned} \tau _e {\dot{E}}&= - E + (1 {-} r_e E) f_e(w_{ee} E {-} w_{ei} I {+} Q_e),\!\! \end{aligned}$$

10b $$\begin{aligned} \tau _i {\dot{I}}&= - I + (1 {-} r_i I) f_i(w_{ie} E {-} w_{ii} I {+} Q_i), \end{aligned}$$

where $E$ and $I$ are the average firing rates of the populations, $\tau _e$ and $\tau _i$ are time constants, $r_e$ and $r_i$ are the refractory periods of neurons, $f_e$ and $f_i$ are functions that describe the response of both populations, $Q_e$ and $Q_i$ are external inputs, and $w_{JK}$ are nonnegative coefficients that describe the links between the populations.

To obtain these equations, Wilson and Cowan use a time coarse graining that leads to see the refractory fraction of a population as proportional to its active fraction. Remark that in our model, such a reduction amounts to fixing each refractory fraction  to its equilibrium solution in the system (8):Using this simplification, the system (8) simply becomes11and is completely equivalent to Wilson–Cowan’s in the case of a pair of populations. Indeed, we can introduce , which is the average proportion of active neurons per unit time—that is, the firing rate—of population $J$. Then, the above equation leads towhere we can write . The parallel with Wilson–Cowan’s original model (10) is then quite clear if we set $\tau _J = \nicefrac {1}{\beta _J}$, $r_J = \nicefrac {1}{\beta _J} + \nicefrac {1}{\gamma _J}$, $f_J = \alpha _J F_J$ and $w_{JK} = \nicefrac {c_{JK}}{\beta _K}$.

The above arguments show that Wilson–Cowan’s dynamical system can be seen as the subsystem (11) of the mean-field system (8), which corresponds to the domainIt is then justified to ask whether the reduction of the system (8) to the subsystem (11) leads to a loss of richness in the dynamics.

A first hint that dynamical behaviors can be lost by the reduction of (8) to the subsystem (11) is given in the following proposition.

### Proposition 2

The domain ${\mathscr {D}}_{n}^\textsc {wc}$ is not invariant by the flow of the dynamical system (8).

### Proof

Let $f: {\mathscr {D}}_{n} \rightarrow {\mathbb {R}}^{2n}$ denote the vector field corresponding to the differential equation (8). Notice that ${\mathscr {D}}_{n}^\textsc {wc}$ is a subset of an euclidean subspace of dimension $n$ in ${\mathbb {R}}^{2n}$. Thus, for ${\mathscr {D}}_{n}^\textsc {wc}$ to be invariant by the flow of (8), $f$ must be tangent to ${\mathscr {D}}_{n}^\textsc {wc}$ at each of its points. However, this cannot be the case, because on ${\mathscr {D}}_{n}^\textsc {wc}$, $f$ has no component along the axes associated with the refractory fractions, whereas ${\mathscr {D}}_{n}^\textsc {wc}$ is not orthogonal to these axes.

**Fig. 2:**
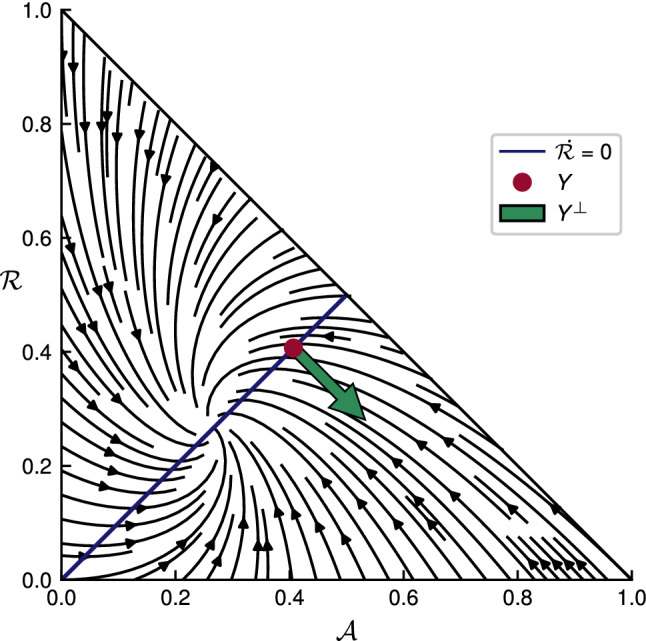
Example of phase plane of the mean-field system (8) in a case of a single population, to illustrate the proof of Proposition 2. Note that the nullcline  is the set ${\mathscr {D}}_{1}^\textsc {wc}$

To be more explicit, fix a population $K\in {\mathscr {P}}$, and choose $x \in (0,1)$ such that

$$\begin{aligned} \frac{\alpha _K\gamma _K}{\alpha _K\beta _K + \alpha _K\gamma _K + \beta _K\gamma _K}< x < \frac{\gamma _K}{\beta _K + \gamma _K}, \end{aligned}$$

which is always possible since $\alpha _K, \beta _K, \gamma _K > 0$. Now, define the vectors  and  by settingand then  for all $J \ne K$. Then $Y \in {\mathscr {D}}_{n}^\textsc {wc}$ and $Y^\perp$ is orthogonal to ${\mathscr {D}}_{n}^\textsc {wc}$. An example of a phase plane with $Y$ and $Y^\perp$ is shown in Fig. 2, in a simple case where the network has a single population.

**Fig. 3:**
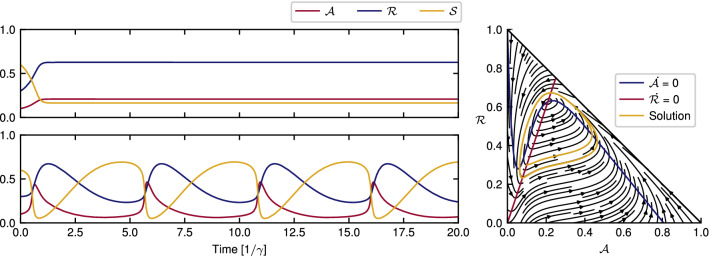
Solutions of the dynamical systems (8) (bottom left) and (11) (top left) with parameters (15) from the initial state (16), and phase plane of the system (8) (right) with the same parameters. The solution illustrated on the phase plane is the same solution as that on the bottom left panel

Suppose $f$ is tangent to ${\mathscr {D}}_{n}^\textsc {wc}$ at $Y$. Then, $f(Y)$ must be orthogonal to $Y^\perp$. Now, all components of $Y^\perp$ are zero except along the axes of population $K$, and  because $Y \in {\mathscr {D}}_{n}^\textsc {wc}$. Thus, . Since $F_K$ is nonnegative, we can easily compute the estimateBy our choice of $x$, it follows that $\langle f(Y),Y^\perp \rangle < 0$, which contradicts the requirement for $f(Y)$ to be orthogonal to $Y^\perp$. Hence, $f$ is not tangent to ${\mathscr {D}}_{n}^\textsc {wc}$ at $Y$, so ${\mathscr {D}}_{n}^\textsc {wc}$ is not invariant by the flow of the dynamical system (8). $\square$

Since an attracting set is invariant by definition, Proposition 2 directly implies the following corollary.

### Corollary

The domain ${\mathscr {D}}_{n}^\textsc {wc}$ is not an attracting set of the dynamical system (8), so its subsystem (11) is not stable.

Proposition 2 and its corollary give information about the relationship between the solutions of the mean-field system (8) and its Wilson–Cowan subsystem (11). If any solution of the mean-field system did eventually converge to a solution of the Wilson–Cowan subsystem—which would happen if ${\mathscr {D}}_{n}^\textsc {wc}$ was an attracting set from ${\mathscr {D}}_{n}$—then we would expect that both models actually lead to the same predictions. However, the corollary to Proposition 2 shows that this is not true, so that we can expect to see different long-term behaviors predicted by the two models, at least in some cases. In fact, Proposition 2 shows that in general, a solution of the Wilson–Cowan subsystem is not even a solution of the mean-field system. This means that if a solution of the mean-field system meets the condition that  for all populations $J$ at some time $t$, this condition does not need to remain true afterward, and the behavior predicted from that point by the Wilson–Cowan subsystem might not be the same as the behavior predicted by the mean-field system.

Nevertheless, the systems (8) and (11) share some properties: for instance, it is easy to see that their fixed points are exactly the same, since the only way for  is that . However, it is not clear at all if the stability of these fixed points remains the same. In fact, we will see in the next section that it is not always the case.

An important difference between these two models is the disparity in the dimension of the dynamical system for the same number of populations. Indeed, in order to model the dynamics of a network of $n$ neural populations, our mean-field system uses a system of $2n$ equation whereas the Wilson–Cowan system only uses $n$. In particular, this means that our mean-field system has enough dimensions to allow oscillations in the activity of a single neural population, unlike the Wilson–Cowan system. Indeed, we will see in Sect. 4.1 an example of an excitatory population whose activity oscillates due to the refractory period. However, it is still not possible with our model to predict oscillations in the activity of a single inhibitory population.

### Proposition 3

In the case of a single population, if $\beta + \gamma > \alpha c \sup F'$, then the mean-field system (8) has no cycles in the domain ${\mathscr {D}}_{1}$.

### Remark

Since the transition rates $\alpha$, $\beta$ and $\gamma$ are positive and $F'$ is nonnegative, the hypothesis of Proposition 3 is always satisfied for a single inhibitory population, since in that case $c < 0$.

### Proof

Let $f : {\mathscr {D}}_{1} \rightarrow {\mathbb {R}}^2$ denote the vector field corresponding to the mean-field system (8), so thatDirect calculations show thatand thatSince $F$ takes its values between $0$ and $1$, it follows that12Now, recall that $\alpha , \beta , \gamma > 0$, that  since we assume that the state , and that $F$ is increasing. Therefore, the divergence (12) is always negative when $c \le 0$. On the other hand, when $c > 0$,which is always negative provided that $\beta + \gamma > \alpha c \sup F'$. As stated in the remark above, this condition includes the case where $c \le 0$. Thus, if $\beta + \gamma > \alpha c \sup F'$, then the divergence of the vector field is always negative in ${\mathscr {D}}_{1}$, and the criterion of Bendixson (1901) guarantees that there are no cycles in ${\mathscr {D}}_{1}$. $\square$

In the same way, the Wilson–Cowan system cannot predict chaotic behavior with two populations, since it has only two dimensions while existence of chaotic solutions requires at least three dimensions in continuous dynamical systems. However, there is no dimensional argument to rule out this possibility with our mean-field system, since it has four dimensions for two populations. We will indeed see in Sect. 4.2 an example of a pair of two excitatory populations that exhibit chaotic behavior.

To further compare the mean-field system and its Wilson–Cowan subsystem, we can add an extra parameter $\varepsilon$ to the mean-field system (8), and consider the dynamical system 13a13b The parameter $\varepsilon$ can then be used to study the transition between the models. First, the system (8) corresponds to the case $\varepsilon = 1$. Then, in the regime where $0 < \varepsilon \ll 1$, (13) is a slow-fast system with two time scales, where the active fractions of populations are the slow variables, whereas the refractory fractions are the fast variables. Ultimately, in the limit where $\varepsilon$ goes to zero, the fast components can be considered to be at equilibrium, so that each refractory fraction is forced to , and we retrieve the Wilson–Cowan subsystem (11). This suggests that the reduction of the mean-field system to the Wilson–Cowan subsystem is valid when the refractory fractions of populations vary much faster than their active fractions, which is the case when the firing rates of populations are small compared to the rates $\beta _J$ and $\gamma _J$. However, this approximation is no longer valid for regimes of larger firing rates where the activation can occur on a time scale comparable to the transitions in and out of the refractory state. It follows that our model can be seen as an extension of the Wilson–Cowan model that is still valid when the firing rate cannot be taken as tending to 0.

## Examples

In this section, we present three examples where the dynamical behavior of the mean-field system (8) is different than that of the Wilson–Cowan subsystem (11), where refractory fractions of populations are fixed to their equilibrium solutions. In all cases, we also show a sample trajectory of the Markov chain described in Sect. 2.1.1. These examples show that there are cases in which the refractory fractions of populations are needed to get an accurate picture of the average behavior of the dynamics on the network.

For all examples presented here, we will assume that the neurons’ activation rate is sigmoidal, with

14 $$\begin{aligned} F_J(y) \,{:}{=}\,\frac{1}{1 + \exp \Bigl ( - \dfrac{y - \theta _J}{s_{\theta _J}} \Bigr )}, \end{aligned}$$

where $\theta _J \in {\mathbb {R}}$ is a threshold parameter and $s_{\theta _J} > 0$ is a scaling parameter.

### A single excitatory population

Consider a network of $N$ neurons with a single population, with parameters

15 $$\begin{aligned} \begin{aligned} \alpha&= 12.5\>[\gamma ],&\theta&= 2, \\ \beta&= 3\>[\gamma ],&s_\theta&= 0.4, \\ \gamma&= 1\>[\gamma ],&Q&= 0, \\ c&= 8, \\ \end{aligned} \end{aligned}$$

where we have dropped subscripts that would refer to the unique population, and where transition rates $\alpha$, $\beta$ and $\gamma$ are measured in units of $\gamma$. Indeed, each term in the equations of the mean-field system (8) is proportional to one of these rates, so setting $\gamma = 1$ is equivalent to measuring time in units of $\nicefrac {1}{\gamma }$. We fix an initial state16Notice that , so that  and the initial state belongs to the domain ${\mathscr {D}}_{1}^\textsc {wc}$.

The mean-field system (8) and its subsystem (11) can be integrated numerically from the initial state (16) with the parameters (15). This yields the solutions shown in Fig. 3. According to Wilson–Cowan’s model, the network’s state converges to a stable fixed point. However, according to our mean-field model where the refractory state is explicitly included, the network’s state rather converges to a limit cycle. We remark that this cycle is rather robust with respect to the values of the transition rates, which is discussed in detail in “Appendix B.”

**Fig. 4:**
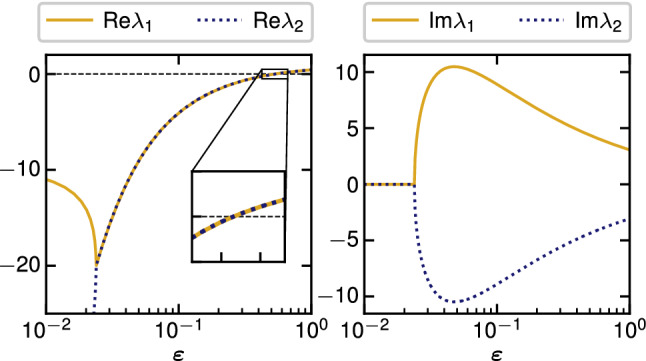
Eigenvalues of the Jacobian matrix of the mixed system (13) with parameters (15) with respect to $\varepsilon$, evaluated at the fixed point to which the Wilson–Cowan subsystem converges

The discrepancy between the long-term behaviors of the mean-field system (8) and the Wilson–Cowan system (11) shown in Fig. 3 suggests that the mixed system (13) undergoes a supercritical Hopf bifurcation as $\varepsilon$ varies from $0$ to $1$, at the fixed point to which the solution of the Wilson–Cowan system converges. This can be verified numerically by computing the eigenvalues of the Jacobian matrix of the system (13) with respect to $\varepsilon$. The results are shown in Fig. 4.

**Fig. 5:**
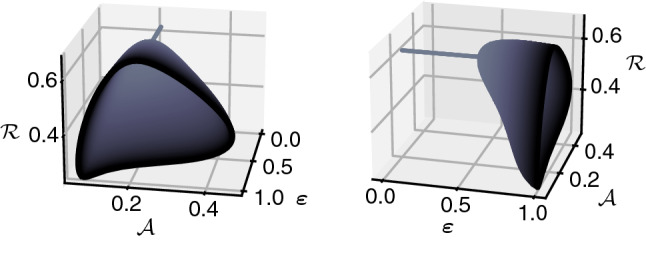
Two views of the three-dimensional bifurcation diagram for the system (13) with parameters (15), where the color of the surface is a function of $\varepsilon$ to make the surface easier to see

To obtain a better understanding of the bifurcation, it is instructive to draw a bifurcation diagram. It is possible to obtain a numerical estimate of such a diagram in the $\varepsilon$–– space. To do so, we first compute the coordinates of the fixed point simply by computing the zero of equation (11) for the parameters (15). Then, for multiple values of $\varepsilon$, we add a small perturbation to the coordinates of the fixed point, and we integrate numerically the mixed system (13). When a stable solution has been reached (either the same fixed point, or a limit cycle around it), we find its coordinates. Plotting these solutions in the $\varepsilon$–– space yields the three-dimensional bifurcation diagram shown in Fig. 5.

**Fig. 6:**
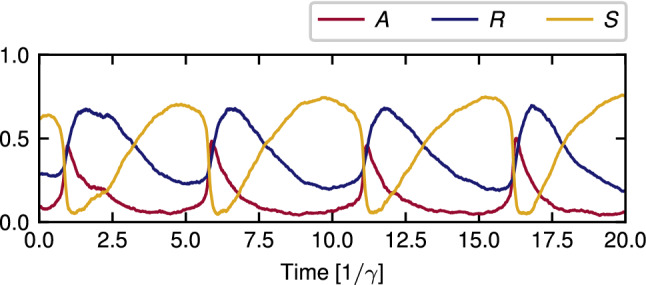
Typical trajectory of the Markov chain described in Sect. 2.1.1 with parameters (15), from an initial state taken randomly such that the probabilities for a neuron to be active and refractory at time zero are respectively $0.1$ and $0.3$

Finally, it is interesting to compare the solutions illustrated in Fig. 3 to sample trajectories of the Markov chain that both macroscopic models seek to approximate. To provide a useful comparison, the same parameters (15) can be used with weights $W = \nicefrac {c}{N}$ between each pair of neurons, and the initial state can be taken randomly so that a neuron is active at time zero with probability $0.1$ and refractory with probability $0.3$. In this way, the microscopic initial state corresponds to (16). Sample trajectories can be obtained from numerical simulations using the Doob–Gillespie algorithm (Gillespie 1976). A typical trajectory obtained with a network of $N = 2000$ neurons is given in Fig. 6, where we clearly distinguish oscillations in the network’s activity that are analogous to those shown in Fig. 3 (bottom). Therefore, we conclude that in this case, the model (8) provides a more accurate prediction of the network’s activity than the Wilson–Cowan subsystem (11).

### An excitator–excitator pair

The last example raises an interesting question: if the activities of two excitatory populations can oscillate by themselves, what happens when they are connected together? We give here a possible answer to this question in a case where two such populations are weakly connected to one another.

Consider a network of $N$ neurons with two excitatory populations, with parameters

17a $$\begin{aligned} \begin{aligned} \alpha _1&= 12.5\>[\gamma _1],&\theta _1&= 2, \\ \beta _1&= 3\>[\gamma _1],&s_{\theta _1}&= 0.4, \\ \gamma _1&= 1\>[\gamma _1],&Q_1&= 0, \end{aligned} \end{aligned}$$

and

17b $$\begin{aligned} \begin{aligned} \alpha _2&= 3.6\>[\gamma _1],&\theta _2&= 0.84, \\ \beta _2&= 8\>[\gamma _1],&s_{\theta _2}&= 0.2, \\ \gamma _2&= 0.8\>[\gamma _1],&Q_2&= 0, \end{aligned} \end{aligned}$$

where in the same way as in the last example, we measure transition rates in units of $\gamma _1$ so that time is measured in units of $\nicefrac {1}{\gamma _1}$. The connections between these populations are described by the matrix

17c $$\begin{aligned} c = \begin{pmatrix} 8 &{} 0.6 \\ 0.01 &{} 14 \end{pmatrix}. \end{aligned}$$

We fix an initial state18Notice that for both populations, , so the initial state belongs to the domain ${\mathscr {D}}_{2}^\textsc {wc}$.

**Fig. 7:**
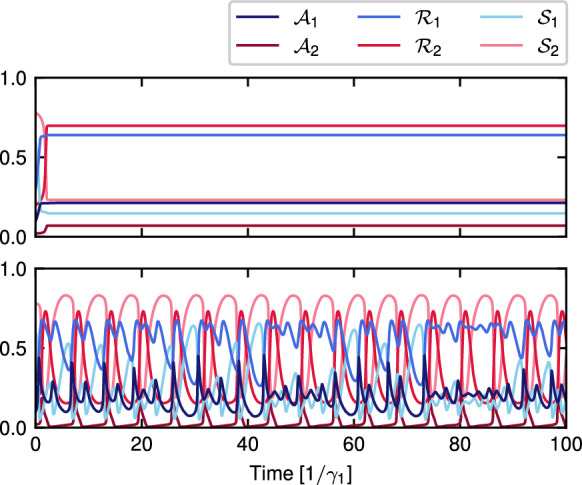
Solutions of the dynamical systems (8) (bottom) and (11) (top) with parameters (17) from the initial state (18)

Integrating numerically the mean-field system (8) and its Wilson–Cowan subsystem from the initial state (18) with the parameters (17) yields the solutions shown in Fig. 7. According to Wilson–Cowan’s model, the network’s state simply converges to a stable fixed point. However, our mean-field model predicts that the network’s activity will exhibit aperiodic behavior, seemingly chaotic.

**Fig. 8:**
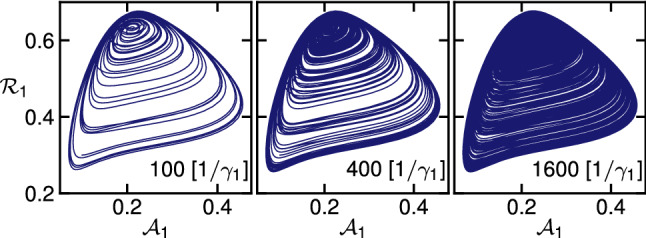
Projections of solutions of the dynamical system (8) with parameters (17) from the initial state (18) over three increasing time intervals

**Fig. 9:**
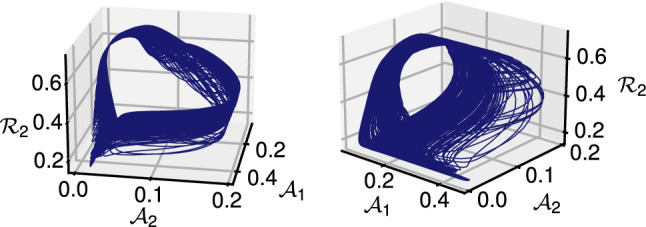
Two views of a projection of the solution of the dynamical system (8) with parameters (17) from the initial state (18) over 1600 time units

To understand the behavior of the network’s state, it is instructive to illustrate the solutions of the mean-field system in other ways. In Fig. 8, the solution of the mean-field system over increasing time intervals is illustrated in the – subspace. The projection of the solution onto this subspace appears not to converge to a point nor to a closed curve, and seems rather to be dense in a bounded subset of the plane. The solution can also be projected onto three-dimensional subspaces. A projection onto the –– subspace is shown in Fig. 9. The solution then seems to converge to a bounded subset of lower dimension, which suggests the presence of a strange attractor.

**Fig. 10:**
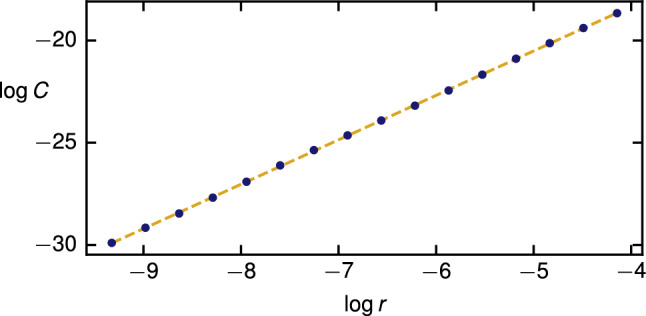
Logarithm of the correlation $C$ as a function of the logarithm of the radius $r$, to estimate the correlation dimension of the attractor

It is then interesting to estimate a fractal dimension for this attractor. Using the method of Grassberger and Procaccia (1983), we estimate its correlation dimension from a solution integrated over 500,000 time units. To do so, we cut the first 1000 time units from the solution to make sure to keep only points on the attractor. Then, we fix a small radius $r$, and for a point $x$ in the remaining points, we count the number $N_x$ of other points lying in a ball of radius $r$ around $x$. We do so for 100 such points $x$, and we average the resulting counts $N_x$ to find a correlation $C(r)$. Applying this recipe for many different values of $r$ between $10^{-4}$ and $10^{-2}$, we obtain a power relation of the form

19 $$\begin{aligned} C(r) \sim r^\nu , \end{aligned}$$

where $\nu$ is the correlation dimension of the attractor. The result is illustrated in Fig. 10. To find a value for $\nu$, we perform a linear regression for $\log C$ as a function of $\log r$. The slope of the fitted line is the correlation dimension. We obtain

20 $$\begin{aligned} \nu = 2.173 \pm 0.005, \end{aligned}$$

the error being the standard error from the linear regression.

To confirm the chaotic behavior of the solution, we compute the largest Lyapunov exponent of the solution, which is a standard method used to either detect or define chaos (Kinsner 2006; Hunt and Ott 2015). We use the discrete QR method as described by Dieci et al. (1997). We do so for four different combinations of integration intervals and time steps. The results are given in Table 1. The largest Lyapunov exponent of the system is positive, indicating a chaotic behavior.

**Table 1:** Values of the largest Lyapunov exponent for numerical solutions of the dynamical system (8) with parameters (17) and initial state (18), for four different combinations of integration interval and time step

Integration interval	Time step	Lyapunov exponent
$[\nicefrac {1}{\gamma _1}]$	$[\nicefrac {1}{\gamma _1}]$	
1000	0.01	0.1592
10,000	0.01	0.1572
1000	0.001	0.1691
10,000	0.001	0.1633

**Fig. 11:**
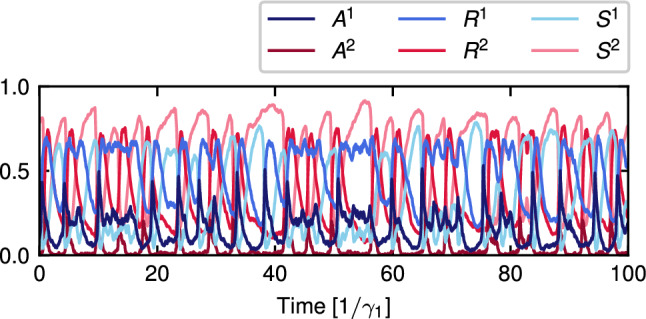
Typical trajectory of the Markov chain described in Sect. 2.1.1 with parameters (17), from an initial state taken randomly so that the probabilities for a neuron to be active or refractory correspond to the macroscopic initial state (18)

Finally, we compare the solutions illustrated in Fig. 7 to sample trajectories of the Markov chain to determine how well the macroscopic models approximate the behavior of the network. To do so, we use the parameters (17) with weights $W_{JK} = \nicefrac {c_{JK}}{|K|}$ from the neurons of population $K$ to neurons of population $J$. We choose randomly an initial state to each neuron of population $J$ so that it is active with probability  and refractory with probability , where the macroscopic initial values are taken from the initial state (18). As is in the first example, sample trajectories are obtained using the Doob–Gillespie algorithm. A typical result for a network of $N = 2000$ neurons, with $1000$ neurons in each population, is given in Fig. 11. As seen on this figure, the evolution of the network’s state does exhibit the aperiodic behavior predicted by our mean-field model. Therefore, we conclude that in this case, including the refractory state explicitly in the dynamical system leads to a more accurate prediction of the network’s activity than to force it to its equilibrium solution.

### An excitator–inhibitor pair

In this last example, we show that the benefit of including the refractory state explicitly in the dynamical system is not only to allow for new dynamical behaviors, but also to predict more accurately the dynamical behavior of the underlying Markov chain in other situations.

Consider a network of $N$ neurons with two populations: one excitatory labeled $E$, with parameters

21a $$\begin{aligned} \begin{aligned} \alpha _E&= 10\>[\gamma _I],&\theta _E&= 0, \\ \beta _E&= 0.8\>[\gamma _I],&s_{\theta _E}&= 0.4, \\ \gamma _E&= 4\>[\gamma _I],&Q_E&= 0, \end{aligned} \end{aligned}$$

and one inhibitory labeled $I$, with parameters

21b $$\begin{aligned} \begin{aligned} \alpha _I&= 9\>[\gamma _I],&\theta _I&= 3, \\ \beta _I&= 1\>[\gamma _I],&s_{\theta _I}&= 0.4, \\ \gamma _I&= 1\>[\gamma _I],&Q_I&= 0, \end{aligned} \end{aligned}$$

where we measure transition rates according to $\gamma _I$ so that time is measured in units of $\nicefrac {1}{\gamma _I}$. The connections between these populations are described by the matrix

21c $$\begin{aligned} c = \begin{pmatrix} c_{EE} &{} c_{EI} \\ c_{IE} &{} c_{II} \end{pmatrix} = \begin{pmatrix} 8 &{} -12 \\ 9 &{} -2 \end{pmatrix}. \end{aligned}$$

We fix an initial state22which belongs to the domain ${\mathscr {D}}_{2}^\textsc {wc}$.

**Fig. 12:**
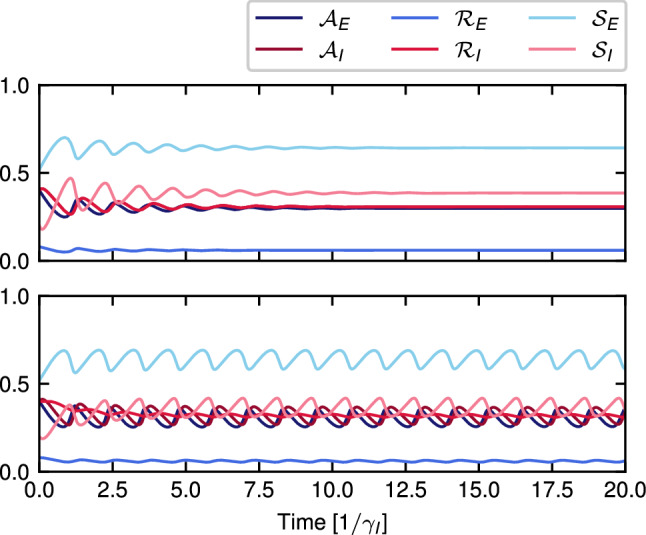
Solutions of the dynamical systems (8) (bottom) and (11) (top) with parameters (21) from the initial state (22)

Integrating numerically the mean-field system (8) and its subsystem (11) with the parameters (21) from the initial state (22) yields the solutions presented in Fig. 12. According to Wilson–Cowan’s model, the network’s state converges to a stable fixed point, but according to our mean-field model, it rather converges to a limit cycle. However, we remark that Wilson–Cowan’s model can also predict oscillations with parameters close to those chosen here: for example, if the connection matrix is changed to $c = \bigl ({\begin{matrix} 9 &{} -12 \\ 9 &{} -1 \end{matrix}}\bigr )$, then both models predict oscillations. Hence, expanding Wilson–Cowan’s model to our mean-field model modifies the values of parameters for which oscillations are predicted.

As in the first example, the difference between the long-term behaviors of the mean-field system and its Wilson–Cowan subsystem suggests a bifurcation with respect to $\varepsilon$ in the mixed system (13). This is verified numerically by computing the eigenvalues of the Jacobian matrix of the mixed system with respect to $\varepsilon$. Doing so indeed shows that as $\varepsilon$ goes from 0 to 1, the real part of a pair of conjugate eigenvalues goes from negative to positive. Hence, we see that the mixed system undergoes a Hopf bifurcation in this interval.

**Fig. 13:**
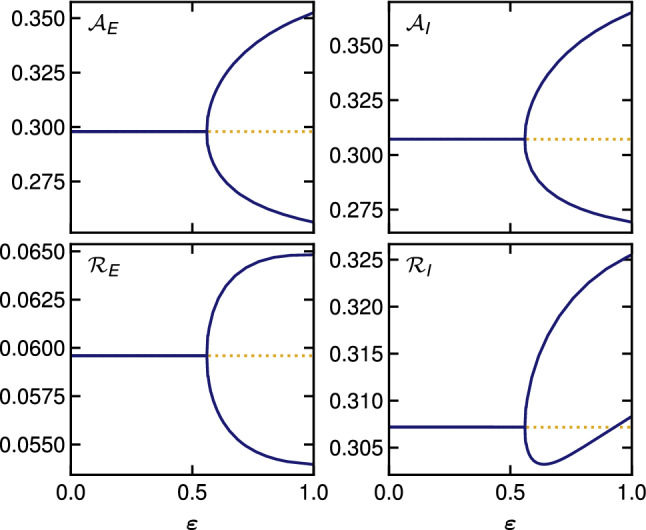
Bifurcation diagrams of the mixed system (13) with parameters (21), where the dotted yellow lines represent the coordinates of the fixed point and the blue lines represent either the maximum and minimum values of the component on the cycle, or the value of the component at the fixed point

It is possible to further understand this bifurcation by drawing a bifurcation diagram. It is unfortunately not possible to draw a complete bifurcation diagram due to the dimension of the system, but it is still possible to obtain a diagram for each individual state component. The result is shown in Fig. 13, where the maximum and minimum values of each state component on the cycle or on the fixed point is plotted against $\varepsilon$. According to the results, the Hopf bifurcation appears to be supercritical.

**Fig. 14:**
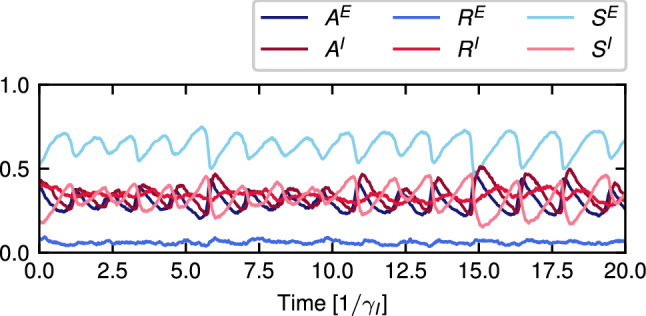
Typical trajectory of the Markov chain described in Sect. 2.1.1 with parameters (21), from an initial state taken randomly such that the probabilities for a neuron to be active or refractory at time zero correspond to the macroscopic initial state (22)

Finally, the solutions of the dynamical systems can be compared to trajectories of the Markov chain whose macroscopic behavior is approximated by both models. To do so, the same parameters (21) can be used with weights $W_{JK} = \nicefrac {c_{JK}}{|K|}$ from neurons of population $K$ to neurons of population $J$. Then, the initial state of each neuron of population $J$ is taken randomly so that it is active with probability  and refractory with probability , where macroscopic initial values are those given in the initial state (22). As in the other examples, sample trajectories are obtained using the Doob–Gillespie algorithm. A typical trajectory with a network of $N = 2000$ neurons with $1000$ neurons in each population is given in Fig. 14. This trajectory exhibits distinct oscillations in the network’s activity. Thus, it is clear that in this case, the mean-field model provides a more accurate approximation of the network’s behavior than its Wilson–Cowan subsystem.

## Conclusion

The Wilson–Cowan model has played an important role in the description of neural systems at the macroscopic level. It has been shown that when considering several excitatory and inhibitory populations, the Wilson–Cowan model can exhibit rich dynamics such as oscillations, bistability and chaos. Furthermore, it is a useful tool in better understanding biological neural networks, especially sensory systems.

One of the assumptions on which the Wilson–Cowan model relies is that the ratio of the numbers of active and refractory neurons is constant in a single population. In this work, we showed that lifting this assumption can reveal novel dynamics in the model, such as oscillations in the activity of a single population or chaotic behavior in the activity of two populations.

An interesting byproduct of our method is that when constructing the model as done in Sect. 2.2, the way in which the dynamics might be affected by correlations between the activities of different populations becomes quite clear. Indeed, to obtain a closed dynamical system, we chose to neglect covariances in equations (7), which led us to approximate the expectations ${{\mathbb {E}}}\big [{F_J(B_J^t)S_t^J}\big ]$ by the corresponding functions where the variables $S_t^J$ and $B_t^J$ are replaced with their expectations. Using a higher-order moment closure, we could take into account the correlations between activities of different populations. We intend to investigate this in future work.

## Figures and Tables

**Fig. 15:**
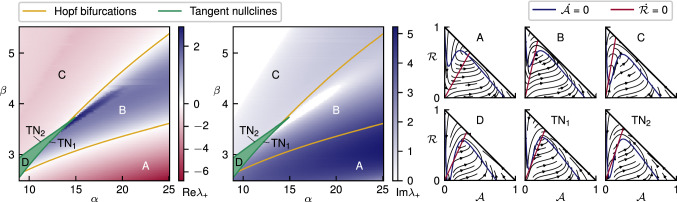
Bifurcation diagram on the parameters $\alpha$ and $\beta$ for the system (8) with parameters (15). In regions A, B and C of the left panels, the colors represent the real and imaginary parts of the eigenvalue $\lambda _+$ of the Jacobian matrix of the system evaluated at the unique fixed point, where $\lambda _+$ is the eigenvalue with maximal real or imaginary part. The abbreviation TN stands for “tangent nullclines.” The right panels give sketches of the phase plane in different regions of the bifurcation diagram

## Data Availability

All numerical results presented in this paper were obtained with the PopNet package (Painchaud 2022), written in the Python programming language and available on GitHub.

## Appendix A: Alternative construction of the model

In this section, we provide an alternative construction of the model presented in Sect. 2, in which we allow parameters to be random, independent and identically distributed over populations. As long as we keep the mean-field assumption, the resulting macroscopic model is still the dynamical system (8). This construction is presented in greater detail and for a more general Markov chain in chapter 2 of (Painchaud 2021); the special case of the Markov chain presented here is discussed in Sect. 4.2 of the latter reference. For basic definitions and results about stochastic processes and Markov chains, we refer to the books by Doob (1990) and Norris (1997).

We start from a similar setting as that described at the beginning of Sect. 2. We consider a network of $N$ neurons labeled from integers from 1 to $N$, and split into a collection ${\mathscr {P}}$ of populations, which are assumed to be made of a large number of neurons. The weight matrix $W$ is now random, defined on a probability space $(H, {\mathscr {H}}, \mu )$, where ${\mathscr {H}}$ and $\mu$, respectively, are a $\sigma$-algebra and a probability measure on the set $H$. We still interpret links between neurons as being either excitatory or inhibitory according to the sign of the corresponding entry of $W$. Then, for each neuron $j$, we consider real-valued random variables $\alpha _j$, $\beta _j$, $\gamma _j$ and $\theta _j$, defined on $(H,{\mathscr {H}},\mu )$ as well, the first three being nonnegative. We assume that all parameters $\alpha _j$, $\beta _j$, $\gamma _j$, $\theta _j$ and $W_{jk}$ are independent random variables, and that they are identically distributed over populations in ${\mathscr {P}}$.

We still see the parameters $\beta _j$ and $\gamma _j$ as being the transition rates of neuron $j$ as in Fig. 1. For the reduction to the macroscopic model to be possible, we will use a different activation rate:

where  is the indicator function of the set

$$\begin{aligned} T_j(x) \,{:}{=}\,\Bigl \{ \eta \in H : \sum _{k=1}^N W_{jk}(\eta ) \mathrm{Re}x_k + Q_J > \theta _j(\eta ) \Bigr \}, \end{aligned}$$

where the external input $Q_J$ is still assumed to be constant over population $J$.

Now, any $\eta \in H$ fixes a choice of parameters, allowing to construct a Markov chain as in Sect. 2.1.1. To do so, we define the matrix $M^\eta \,{:}{=}\,\{m^\eta (x,y) : x,y \in E\}$ with entries

$$\begin{aligned} m^\eta (x,y) \,{:}{=}\,\sum _{j=1}^N m_j^\eta (x,y) \prod _{\begin{array}{c} k=1 \\ k\ne j \end{array}}^N \delta _{x_ky_k} \end{aligned}$$

where

$$\begin{aligned} \begin{aligned} m_j^\eta (x,y) \,{:}{=}\,a_j(\eta , x)&(1 - |x_j|) \bigl ( \mathrm{Re}y_j - (1 - |y_j|) \bigr ) \\ {} + \beta _j(\eta )&\mathrm{Re}(x_j) (\mathrm{Im}y_j - \mathrm{Re}y_j) \\ {} + \gamma _j(\eta )&\mathrm{Im}(x_j) \bigl ( (1 - |y_j|) - \mathrm{Im}y_j \bigr ). \end{aligned} \end{aligned}$$

Again, $M^\eta$ is the generator of a continuous-time Markov chain. Hence, it follows from the Kolmogorov extension theorem that a probability measure ${\mathbb {P}}^\eta$ exists on $(\Omega , {\mathscr {F}}) \,{:}{=}\,(E,2^E)^{[0,\infty )}$ such that for any $x,y \in E$, as $\Delta t \downarrow 0$,

$$\begin{aligned} {{\mathbb {P}^\eta }}[{X_{t+\Delta t}=y \vert X_t=x}] = \delta _{xy} + m^\eta (x,y) \Delta t + o(\Delta t), \end{aligned}$$

where $\{X_t\}_{t\ge 0}$ is the coordinate mapping process on $(\Omega ,{\mathscr {F}})$, defined as $X_t(\omega ) \,{:}{=}\,\omega (t)$. Similar relations to (1) with a dependence on $\eta$ also hold. The measures ${\mathbb {P}}^\eta$ can be combined in a single measure ${\mathbb {P}}$ on the product space $(H \times \Omega , {\mathscr {H}} \otimes {\mathscr {F}})$ such that

23 $$\begin{aligned} {{\mathbb {E}}}\big [{Z}\big ] = \int _H \int _\Omega Z(\eta ,\omega ) {\text {d}}{}\!{\mathbb {P}}^\eta (\omega ) {\text {d}}{}\!\mu (\eta ) \end{aligned}$$

for any random variable $Z$ on $(H \times \Omega , {\mathscr {H}} \otimes {\mathscr {F}})$, where ${\mathbb {E}}$ is the expectation with respect to ${\mathbb {P}}$.

To study the average behavior of the Markov chain, we start by defining

$$\begin{aligned} p_j^\eta (t)&\,{:}{=}\,{{\mathbb {P}}}\big [{X_t^j = 1 \vert \eta }\big ], \\ q_j^\eta (t)&\,{:}{=}\,{{\mathbb {P}}}\big [{X_t^j = 0 \vert \eta }\big ], \\ r_j^\eta (t)&\,{:}{=}\,{{\mathbb {P}}}\big [{X_t^j = i \vert \eta }\big ], \end{aligned}$$

where we condition on the projection onto $H$. Then, in the same way as in Sect. 2.1.2, it can be shown that

$$\begin{aligned} {\dot{p}}_j^\eta (t)&= - \beta _j(\eta ) p_j^\eta (t) + {\mathbb {E}}\big [\bigl ( 1 - |X_t^j| \bigr ) a_j(\eta , X_t) \vert \eta \big ], \\ {\dot{q}}_j^\eta (t)&= - {\mathbb {E}}\big [\bigl ( 1 - |X_t^j| \bigr ) a_j(\eta , X_t) \vert \eta \big ] + \gamma _j(\eta ) r_j^\eta (t), \\ {\dot{r}}_j^\eta (t)&= - \gamma _j(\eta ) r_j^\eta (t) + \beta _j(\eta ) p_j^\eta (t). \end{aligned}$$

Now, we can define

$$\begin{aligned} p_j(t)&\,{:}{=}\,{{\mathbb {P}}}\big [{X_t^j = 1}\big ], \\ q_j(t)&\,{:}{=}\,{{\mathbb {P}}}\big [{X_t^j = 0}\big ], \\ r_j(t)&\,{:}{=}\,{{\mathbb {P}}}\big [{X_t^j = i}\big ]. \end{aligned}$$

Then, ${\dot{p}}_j(t) = \frac{{\text {d}}{}\!}{{\text {d}}{}\! t} \int _H p_j^\eta (t) {\text {d}}{}\!\mu (\eta )$. The functions $\eta \mapsto p_j^\eta (t)$ can be dominated by the function , which is integrable over $H$, so that the derivative can be passed under the integral sign. Similar bounds hold for $q_j^\eta (t)$ and $r_j^\eta (t)$, and it follows that

$$\begin{aligned} {\dot{p}}_j(t)&= - {{\mathbb {E}}}\big [{\beta _j \mathrm{Re}X_t^j}\big ] + {\mathbb {E}}\big [\bigl ( 1 - |X_t^j| \bigr ) a_j(\cdot , X_t)\big ], \\ {\dot{q}}_j(t)&= - {\mathbb {E}}\big [\bigl ( 1 - |X_t^j| \bigr ) a_j(\cdot , X_t)\big ] + {{\mathbb {E}}}\big [{\gamma _j \mathrm{Im}X_t^j}\big ], \\ {\dot{r}}_j(t)&= - {{\mathbb {E}}}\big [{\gamma _j \mathrm{Im}X_t^j}\big ] + {{\mathbb {E}}}\big [{\beta _j \mathrm{Re}X_t^j}\big ]. \end{aligned}$$

To pass to the macroscopic point of view, we consider for $J \in {\mathscr {P}}$ the fractions of population $A_t^J$, $R_t^J$ and $S_t^J$ defined as in Sect. 2.2, that is,

$$\begin{aligned} A_t^J&\,{:}{=}\,\frac{1}{|J|} \sum _{j\in J} \mathrm{Re}X_t^j, \\ R_t^J&\,{:}{=}\,\frac{1}{|J|} \sum _{j\in J} \mathrm{Im}X_t^j, \\ S_t^J&\,{:}{=}\,\frac{1}{|J|} \sum _{j\in J} \bigl ( 1 - |X_t^j| \bigr ). \end{aligned}$$

Now that parameters are random, we cannot simply sum over populations to obtain the differential equations (7) from the expressions we obtained for the derivatives of $p_j$, $q_j$ and $r_j$. However, we can still argue that the same macroscopic dynamical system describes the average behavior of the network’s activity, at least approximatively. To do so, we define the subpopulations

$$\begin{aligned} J_t^\xi \,{:}{=}\,\{j \in J : X_t^j = \xi \} \end{aligned}$$

for $\xi \in \{0,1,i\}$. The size of these subpopulations can easily be related to the macroscopic state variables. Indeed, since $\mathrm{Re}X_t^j = 1$ if and only if $X_t^j = 1$, then $|J_t^1| = |J| A_t^J$. Similarly, $|J_t^0| = |J| S_t^J$ and $|J_t^i| = |J| R_t^J$. Now, notice that

$$\begin{aligned} \frac{1}{|J|} \sum _{j\in J} \beta _j \mathrm{Re}X_t^j = \frac{1}{|J|} \sum _{j\in J_t^1} \beta _j = \frac{A_t^J}{|J_t^1|} \sum _{j\in J_t^1} \beta _j. \end{aligned}$$

Assuming that the random variables $\beta _j$ are independent and identically distributed over populations, if the size of $J$ is very large, the law of large numbers leads to expect the average $\frac{1}{|J|} \sum _{j\in J} \beta _j$ to be very close to the expectation $\beta _J \,{:}{=}\,{{\mathbb {E}}}\big [{\beta _j}\big ]$. In the same way, since the value of $\beta _j$ should not influence the probability that $j$ is active at time $t$, we expect the average $\frac{1}{|J_t^1|} \sum _{j\in J_t^1} \beta _j$ to be very close to $\beta _J$. This leads to approximate

24 $$\begin{aligned} \frac{1}{|J|} \sum _{j\in J} \beta _j \mathrm{Re}X_t^j \approx \beta _J A_t^J, \end{aligned}$$

for a large population $J$. In the same way, we approximate

25 $$\begin{aligned} \frac{1}{|J|} \sum _{j \in J} \gamma _j \mathrm{Im}X_t^j = \frac{R_t^J}{|J_t^i|} \sum _{j \in J_t^i} \gamma _j \approx \gamma _J R_t^J \end{aligned}$$

where $\gamma _J \,{:}{=}\,{{\mathbb {E}}}\big [{\gamma _j}\big ]$ for $j \in J$, and

$$\begin{aligned} \sum _{k=1}^N W_{jk} \mathrm{Re}X_t^k = \sum _{K\in {\mathscr {P}}} \sum _{k \in K_t^1} W_{jk} \\ \approx \sum _{K\in {\mathscr {P}}} |K| A_t^K W_{JK} = \sum _{K\in {\mathscr {P}}} c_{JK} A_t^K \end{aligned}$$

where $c_{JK} \,{:}{=}\,|K| W_{JK}$ and $W_{JK} \,{:}{=}\,{{\mathbb {E}}}\big [{W_{jk}}\big ]$ for $j \in J$ and $k \in K$. The last case requires more attention. Using the last approximation, we start by approximating

where $B_t^J \,{:}{=}\,\sum _{K\in {\mathscr {P}}} c_{JK} A_{t}^{K} + Q_{J}$. Using the same argument as in the last cases, we approximate by replacing the rate  with its expectation given the state of the network. Since $\alpha _j$ and $\theta _j$ are independent, we obtain

26

where $\alpha _J \,{:}{=}\,{{\mathbb {E}}}\big [{\alpha _j}\big ]$ for $j \in J$ and where $F_J$ denotes the cumulative distribution function of $\theta _j$ for $j \in J$, which is the expectation of  given the state of the network. We remark that this approximation is based on idea that the set $J_t^0$ is large, and that the approximate activation rate  has no influence on the probability for neuron $j$ to be sensitive at time $t$. However, one could expect this to be false, especially if the standard deviation of $\theta _j$ is high: if the threshold $\theta _j$ is higher, it is harder for $j$ to reach its threshold input and to activate. Therefore, we expect this last approximation to be valid only when the standard deviation of $\theta _j$ is small.

Now, consider the macroscopic state variables ,  and . By linearity of the derivative,

Hence, the approximations (24) and (26) lead to approximate

In the same way, the approximations (24), (25) and (26) lead to

and

Thus, the dynamical system (7) describes approximatively the evolution of the network’s activity. In the same way as in Sect. 2.2, the mean-field assumption yields the dynamical system (8).

We conclude by noticing that even though the approximations we made here seem reasonable, it would be far from obvious to make the argument fully rigorous by properly taking a limit as the sizes of the populations grow infinitely large. This is because the probability measure ${\mathbb {P}}$ defined in (23) depends on the measures ${\mathbb {P}}^\eta$, which are defined from the generators $M^\eta$ and thus depend on the network’s size. Hence, to vary the network’s size, we would need to vary the probability measure, and it is not clear how these different measures can be related. Moreover, the sets over which averages of parameters are approximated to their expectations are subpopulations of neurons that are in a given state at a given time, which are random. Thus, to use correctly some sort of law of large numbers, we would need to ensure that these subpopulations become arbitrarily large with a high probability.

## Appendix B: Further bifurcation analysis of Example 4.1

In this section, we investigate the impact of the transition rates on the dynamics of the single-population network of Example 4.1, with the idea to understand how robust is the limit cycle with respect to changes in their values. As before, we measure all three transition rates $\alpha$, $\beta$ and $\gamma$ in units of $\gamma$, which means that we keep $\gamma = 1$. Thus, we are really only interested in modifying $\alpha$ and $\beta$. We keep the other parameters fixed, with values given by (15).

We start by studying the number of fixed points of the system. We do so from the nullclines

and

An illustration of these nullclines in the case where $\alpha$ and $\beta$ have the values of Example 4.1 is given on the phase plane on the right panel of Fig. 3. The fixed points of dynamical system are the points where these nullclines intersect, so they correspond to the zeros of

Notice that $g(0) = 1$, while

$$\begin{aligned} g\Bigl ( \frac{\gamma }{\beta + \gamma } \Bigr ) = - \frac{\beta \gamma }{\alpha (\beta + \gamma ) F(\frac{c\gamma }{\beta +\gamma } + Q)} < 0. \end{aligned}$$

Thus, the intermediate value theorem shows that the system has at least one fixed point  with . At such a fixed point, the refractory fraction is , and .

Now, assuming that $F$ is given by the sigmoidal function (14), it has the property that

Using this result, it is straightforward to compute

Since $F < 1$, it follows that $g''$ has a single zero. Hence, $g$ has a single inflection point, and it cannot have more than three zeros.

The above discussion shows that the system has between one and three fixed points. It would be hard to determine precisely the location and the number of fixed points analytically, but it can be done numerically. Indeed, for a given value of $\alpha$, it is possible to compute the values of $\beta$ for which the nullclines are tangent to each other. These values of $\beta$ corresponds to the boundary between the domains of the $\alpha$–$\beta$ space where the system admits one and three fixed points. Then, when there is only one fixed point, it is possible to find it numerically, and to compute the eigenvalues of the Jacobian matrix of the system to determine its stability. The result is illustrated in Fig. 15. The regions A, B and C of the bifurcation diagram are those where the system admits a single fixed point, and region D is that where it admits three fixed points. The boundary of region D corresponds to the points where the nullclines are tangent.

Two important conclusions can be understood from these numerical results. First, in region B, the system admits a single fixed point which is unstable, since at least one eigenvalue of the Jacobian matrix of the system has positive real part. Since the domain ${\mathscr {D}}_{1}$ is always invariant, it then follows from the theorem of Poincaré– Bendixson (1901) that a limit cycle exists somewhere in ${\mathscr {D}}_{1}$. Moreover, on the curves that bound region B, where the real part of the eigenvalue $\lambda _+$ is zero, the imaginary part of $\lambda _+$ is nonzero. Hence, on these curves, the eigenvalues of the Jacobian matrix are conjugates, and their real part changes sign. This implies that the system undergoes Hopf bifurcations on these curves.

Therefore, if the values of $\alpha$ and $\beta$ lie in region B in Fig 15, then a limit cycle exists. We remark however that, a priori, this condition is sufficient but not necessary. Indeed, if the Hopf bifurcations on the bounds of region B are subcritical, the limit cycle may very well continue to exist outside of region B. Further analysis would be needed to determine with more precision the values of $\alpha$ and $\beta$ which yield a limit cycle.
